# Methicillin-Resistant *S. aureus* Carrying the PVL and Toxic Shock Syndrome Toxin in Healthy Dogs in Algeria

**DOI:** 10.3390/antibiotics13111090

**Published:** 2024-11-15

**Authors:** Fares Khermouche, Nouzha Heleili, Manel Merradi, Amina Hachemi, Antoine Drapeau, Séverine Murri, Jean-Yves Madec, Marisa Haenni

**Affiliations:** 1 Laboratoire ESPA, Département Vétérinaire, Institut des Sciences Vétérinaires et des Sciences Agronomiques, Université Batna 1, Batna 05000, Algeria; fares.khermouche@univ-batna.dz (F.K.); nouzha.heleili@univ-batna.dz (N.H.); m.merradi@univ-batna2.dz (M.M.); 2 Département de Microbiologie et de Biochime, Faculté des Sciences de la Nature et de la Vie, Université Batna 2, Batna 05078, Algeria; 3 Laboratoire HASAQ, Ecole Nationale Supérieure Vétérinaire, Alger 16000, Algeria; a.hachemi@ensv.dz; 4 Unité Antibiorésistance et Virulence Bactériennes, ANSES—Université de Lyon, 69007 Lyon, France; antoine.drapeau@anses.fr (A.D.); severine.murri@anses.fr (S.M.); jean-yves.madec@anses.fr (J.-Y.M.)

**Keywords:** MRSA, dog, PVL, TSST, Algeria

## Abstract

**Background/Objectives**: *Staphylococcus aureus* and *Staphylococcus pseudintermedius* are major opportunistic pathogens in both humans and dogs. In pets, the dissemination of methicillin-resistant isolates (MRSA or MRSP) is problematic for the treatment of animals and is a public health issue due to their zoonotic potential. MRSA and MRSP may also harbor virulent genes that increase their dangerousness. This study aimed to assess the prevalence of (MR)SA and (MR)SP in healthy dogs and their owners in Algeria. **Methods**: Swabs were collected from various body sites of healthy dogs (n = 88) and from the nose of their owners (n = 38). Antimicrobial susceptibility testing was performed by antibiograms according to the disc diffusion method, and clonality was assessed using Pulsed-Field Gel Electrophoresis (PFGE). All methicillin-resistant isolates were short-read whole-genome sequenced using the Illumina technology. **Results**: 26 *S. aureus* and 17 *S. pseudintermedius* isolates were respectively collected from 13 dogs (13/88, 14.8%). No MRSP isolate was detected, while MRSA was found in six dogs (6.8%). Isolates belonged to ST1 (n = 3), ST 80 (n = 1), and ST 22 (n = 2, including the single-locus variant ST7118). All MRSA displayed the immune evasion cluster (IEC) type E. The ST80 isolate presented the Panton–Valentine toxin, and the ST22/ST7118 isolates carried the *tst* gene coding for the toxic shock syndrome toxin. **Conclusions**: The epidemiology of MRSA in healthy Algerian dogs mirrors the one in Algerian people. This poses a zoonotic and public health concern due to the virulence and resistance genes displayed by these isolates. Our results indicate the need for developing One Health strategies to avoid a large-scale dissemination of MRSA in Algerian dogs.

## 1. Introduction

*Staphylococcus pseudintermedius* is the main coagulase-positive *Staphylococcus* associated with dogs, identified in 37% to 92% of healthy animals and colonizing multiple body sites [1]. A recent meta-analysis reported a carriage rate of 18.3% at the world level, with little variations between countries except for Oceania, which reported a much higher prevalence [2]. When *S. pseudintermedius* carries the *mecA* gene that confers resistance to methicillin and all beta-lactam antibiotics, it also often co-harbors additional resistance genes, making it difficult to treat with the therapeutic arsenal available to veterinarians. Methicillin-resistant *S. pseudintermedius* (MRSP) clones have been reported in Europe, North America, and Asia, often associated with sequence types (ST)71, ST68, and ST45/ST112 [3], but have not yet been widely reported from African countries other than South Africa [4,5,6,7]. Although primarily adapted to dogs [8], (MR)SP can also infect humans, and nosocomial as well as intra-household transmission have been reported [9].

Just as *S. pseudintermedius* is adapted to dogs, *Staphylococcus aureus* is particularly adapted to the human host, where it is both a commensal and an opportunistic pathogen causing mild to severe infections. Methicillin-resistant *S. aureus* (MRSA) presenting the *mecA* gene have disseminated in the human population through waves of clones that were successful in hospital settings (HA-MRSA) or the community (CA-MRSA) [10]. MRSA infecting or colonizing cats and dogs generally belongs to the same clones as those circulating in humans in the same country. For example, the Lyon and Geraldine clones have been reported in France [11], ST8/ST22 in Austria [12], or ST5 in Brazil [13]. As with (MR)SP, the presence of (MR)SA from companion animals on the African continent is still poorly documented [14], but a recent review reported that the prevalence of MRSA in Africa was the highest (7.6%) when compared to all five continents [2].

*S. pseudintermedius* and *S. aureus* are zoonotic pathogens whose study in cats and dogs is of major interest in Africa, not only because of the lack of data, but also because of the increasing importance of pets in these countries. In Algeria, a few studies reported the occurrence of (MR)SA in animals, but mainly in livestock species such as cows [15,16], bovines [17], goats [18], or camels [19,20]; meanwhile, there is a clear gap of knowledge regarding the prevalence, genotypes, and clonal diversity of (MR)SA and (MR)SP in dogs. To the best of our knowledge, only two studies investigated this field. The first one reported 8.6% of *S. pseudintermedius* (with no resistance to methicillin) and the absence of *S. aureus* in a collection of 70 isolates from healthy cats and dogs [21], while the second one reported a 4.3% proportion of MRSA in pets and the presence of ST80 PVL-positive isolates in three cats [22].

Knowing the extent of (MR)SA/(MR)SP colonization of dogs in Algeria and the nature of the clones they carry is crucial for developing infection control approaches adapted to the local context and providing useful data for public health and veterinary medicine from a One Health perspective. Thus, the aim of this study was to determine the proportion of these zoonotic pathogens in healthy dogs and their owners in the Eastern region of Algeria. Swabs taken from various body sites were analyzed for the presence of (MR)SA/(MR)SP, and isolated bacteria were identified to the species level and characterized phenotypically using antimicrobial susceptibility testing by antibiograms. Furthermore, all methicillin-resistant isolates were whole-genome sequenced. The results of this study will deepen our knowledge of the clones, resistance, and virulence genes that are circulating in Algerian pets and could be further disseminated to people and other animals in contact, as well as to the environment of the dogs.

## 2. Results

### 2.1. Prevalence of S. aureus and S. pseudintermedius Isolates

All isolates were identified as *S. aureus* or *S. pseudintermedius* using species-specific primers (see Materials and Methods Section 4.2). *S. aureus* isolates were identified in 13 dogs (13/88, 14.8%) and seven owners (7/38, 18.4%), while *S. pseudintermedius* were found in 13 dogs (14.8%) and two owners (5.3%) (Table S1). A total of 26 *S. aureus* and 17 *S. pseudintermedius* were collected since four dogs (D18, D43, D44, D46) displayed two *S. aureus* isolates, one (D36) presented two *S. pseudintermedius* isolates, one (D13) displayed two *S. aureus* and one *S. pseudintermedius* isolate, and a last one (D17) presented two *S. aureus* and two *S. pseudintermedius* isolates. When two isolates from the same bacterial species originated from the same dog, these isolates were either collected from different sampling sites (nose and ear) or from the left and right ear/nare.

In six households, *S. aureus* and/or *S. pseudintermedius* isolates were collected from two dogs (household 6 and 16), one dog and the owner (household 15), or two dogs and the owner (household 4, 12, and 18). Pulsed-Field Gel Electrophoresis (PFGE) profiles proved that only two dogs (D43 and D44) from the same household shared the same isolate. Whole-genome sequencing showed that this methicillin-susceptible *S. aureus* belonged to ST291 and displayed the *spa*-type t2313. This isolate only carried the *blaZ* resistance gene, as well as the immune evasion cluster (IEC) type E cluster (*sak* and *scn* genes in the absence of the *chp* gene and associated enterotoxins). In all other cases, including the two cases (H9, H21) where an owner presented the same staphylococcal species as one of his dogs, isolates presented different PFGE profiles.

### 2.2. Resistance Phenotypes

Among the 26 *S. aureus* isolates, eight coming from six different dogs (6/88, 6.8%) were resistant to cefoxitin (Table 1). The PCR detection of the *mecA* gene confirmed that these eight isolates were MRSA, and PFGE profiles proved that the two isolates each identified in two dogs (D13 and D18) were clonal. Antibiograms by disc diffusion showed that the eight MRSA isolates were additionally resistant to penicillin G (n = 8), kanamycin (n = 6), tetracycline (n = 6), erythromycin (n = 6), lincomycin (n = 2), spiramycin (n = 1), and fusidic acid (n = 1). Among MSSA, 13 isolates were resistant to penicillin G, one to tetracycline, two to erythromycin, one to fusidic acid, and one to enrofloxacin.

No methicillin-resistant *S. pseudintermedius* was identified among the 17 collected isolates, which were susceptible to most of the antibiotics tested since only 10 isolates (58.8%) were resistant to tetracycline, five (29.4%) to penicillin G, and one to fusidic acid (5.9%).

*S. aureus* isolates were statistically more resistant than *S. pseudintermedius* to penicillin G, kanamycin, erythromyin, spiramycin, lincomycin, and enrofloxacin, while they were statistically less resistant to tetracycline.

### 2.3. Genomic Characterization of MRSA Isolates

One non-clonal isolate per dog (n = 6) was whole-genome sequenced (Table 2). Three isolates belonged to ST1 and displayed either the t127 (n = 2) or the t948 (n = 1) *spa*-type. One isolate belonged to ST80, one to ST22, and the last one to ST7118 (*glpF* single-locus variant (SLV) of ST22). The *mecA* gene was located on an SCC*mec* type IVa(2B) in all isolates, except in the ST80 isolate, which presented the type IVc(2B) variant. Resistance genes identified were coherent with the phenotypes, with four isolates presenting genes conferring resistances to beta-lactams (*blaZ*, *mecA*), aminoglycosides (*ant(6)-Ia*, *aph(3′)-III)*), macrolides (*erm(C)*), and tetracycline (*tet(K)*), with the last two isolates displaying only the *blaZ* and *mecA* genes.

All isolates carried the IEC type E. Moreover, the ST80 isolate presented the Panton–Valentine toxin (PVL), while the ST22 isolate and its SLV ST7118 displayed the *tst* gene coding for the toxic shock syndrome toxin.

### 2.4. Comparison with Algerian Clinical Isolates of Identical Sequence Types

**Figure 1:**
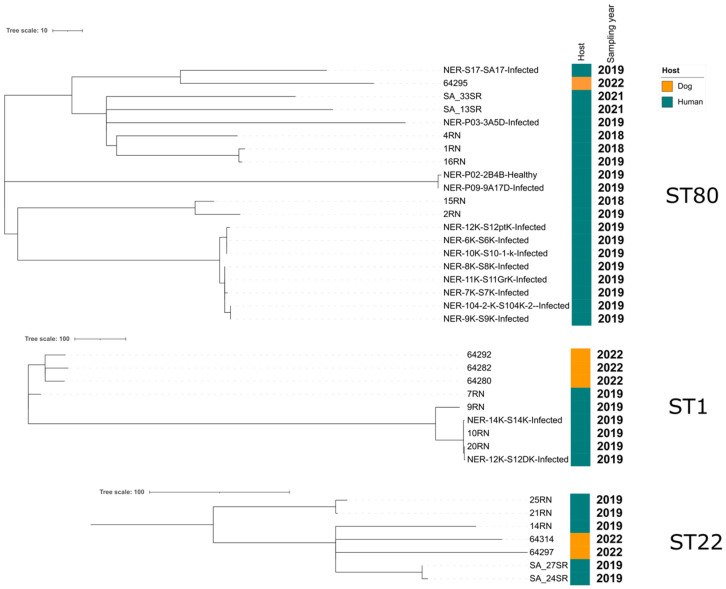
SNP-based phylogeny of the ST1, ST22, and ST80 genomes obtained from Algerian dogs and humans. In the ST22 group, the isolate 64314 corresponds to the single locus variant ST7118.

The genomes from the dog isolates collected in this study were compared to publicly available MRSA genomes retrieved in Algeria (NCBI, last accessed August 2024) and belonging to ST80 (n = 19), ST22 (n = 5) and ST1 (n = 6) (Figure 1)

All retrieved genomes originated from humans. They were collected from hospitalized patients (either from healthy skin or diabetic foot ulcers) in Constantine in 2019 [23], from hospitalized patients or environmental surfaces in Western Algeria in 2020–2021 [24], or from hospitalized patients (MRSA were all retrieved from pus) in the Chlef province (Northern Algeria) in 2018–2019 [25]. A SNP-based phylogeny (Figure 1) showed that dog isolates were not clonally related to human isolates, since all dog genomes differed by more than 10 SNPs from the human genomes (Table S2).

Only a few human genomes collected from the same hospital were clonal. Among dogs, the three ST1s differed by less than 100 SNPs, showing similarities but no clear epidemiological link.

## 3. Discussion

In this study, 14.8% (13/88) of the dogs presented an *S. aureus* isolate, and the same proportion carried an *S. pseudintermedius* isolate. This is similar to what has been recently reported by Abdullahi et al. at a global scale, with isolation rates of 18.3% for *S. pseudintermedius* and 10.9% for *S. aureus* [2], but lower than the carriage rate usually reported for *S. pseudintermedius* (above 35%) in healthy dogs [1]. Methicillin resistance was not identified among *S. pseudintermedius*, while MRSA isolates were found in 6.8% of the dogs. This is the first report of MRSA in healthy Algerian dogs, while this pathogen had been previously reported in goats [2], livestock [2], and poultry [26]. The proportions of MRSA vary depending on the site of sampling (skin, nose, vagina) and the media used for selection. Here, samples were processed on non-selective media, so the proportion of MRSP/MRSA observed is most probably under-estimated, possibly explaining why no MRSP was identified.

Concomitantly to dogs, nasal samples of 38 owners were also tested, and only seven *S. aureus* (18.4%) and two *S. pseudintermedius* (5.3%) were identified, of which none were methicillin-resistant. When one *S. aureus* or one *S. pseudintermedius* was collected from the owner and his dog, PFGE profiles showed that they were not identical, excluding any event of transmission between humans and animals. In our study, intra-household sharing was only observed for two ST291 *S. aureus* isolates found in two dogs. A study performed in Germany identified sharing of methicillin-susceptible *S. aureus* and *S. pseudintermedius* between humans and their dogs, and concluded that sharing of *S. pseudintermedius* is less likely than that of *S. aureus*, as observed here [27]. Usually, intra-household transmission involved MRSA, and mostly community-acquired MRSA (CA-MRSA) that primarily infected the owner [28,29]. This indicates that CA-MRSA might be more prone to disseminate between hosts and in their surrounding environment due to their epidemiological success. However, this might also point out the methodological difficulty of finding identical isolates that cannot be selected based on their resistance phenotype in two different hosts in which they might reside in the sub-dominant flora.

Many studies reported that, when MRSA is identified in cats and dogs, the epidemiology mostly mirrors the one that is reported in humans in the same country [11,12,13]. This is also true in Algeria, where the ST1, ST22, and ST80 identified in dogs are also highly prevalent in human patients. The ST80 clone, a CA-MRSA carrying the PVL toxin, emerged before 2006 in humans and became largely dominant all over the country [23,24,25,30,31,32]. Since then, this clone has been described in nasal samples of sheep and camels [19], in fresh fecal droppings and intestinal content of wild animals and fishes [19], and in unpasteurized milk [33]. Unfortunately, these isolates were not fully sequenced, so we could not compare them with our dog isolate. The isolate collected in this study displayed an IEC type E, which allows *S. aureus* to evade the human immune system, and thus probably suggests the human origin of this pathogen.

ST22 is a highly successful clone causing both hospital- and community-acquired infections in humans across Europe and worldwide [32,34]. It has also been identified in dogs in Germany and in France [11,35], and now in Algeria (one ST22 and one SLV ST7118) carrying the *tst* gene. A *tst*-positive ST22 has already been reported in humans in Algeria [24,25], while only *tst*-negative isolates had been identified in cows and their caretakers [15].

Finally, the ST1 clone, whose proportion is increasing in Africa [32], is also causing both hospital- and community-acquired infections across the world. The three positive MRSA ST1 clones were recovered from two dogs in Sétif and one dog in Batna, both located in the east of Algeria and approximately 150 km apart. This suggests that this clone has been disseminated throughout the country and may be found elsewhere if investigated. The ST1-t127 clone, identified here in two dog samples, is considered the second most common clone globally detected in companion animals, livestock, and livestock products in numerous countries. In Algeria, this clone has been described in MRSA and MSSA from clinical samples [25], but here we report its first description in animals.

The fact that these three clones widely found in Algerian hospitals were those identified in dogs from the same country illustrates the need for a One Health approach to avoid the wider dissemination of these resistant and virulent clones in the human community and in animals. Additionally, the detection of virulence and host adaption systems in *S. aureus* from cattle, wild animals, and now pets represents an important public health issue, since these animals can act as intermittent carriers or reservoirs of zoonoses.

## 4. Materials and Methods

### 4.1. Study Design

Between May 2022 and December 2023, 149 swabs were collected from various body sites (nose (n = 76), ears (n = 69), skin (n = 3), and vagina (n = 1)) of 88 healthy dogs of different ages, breeds, and genders (Table S1). Furthermore, 38 swabs were collected from pet owners’ noses. A veterinarian went door-to-door, swabbing dogs whenever their owners agreed. All dogs came from the cities of Setif (n = 43), Batna (n = 26), Khenchla (n = 15), and Msila (n = 4) in the Eastern region of Algeria. Neither people nor dogs had been treated with antibiotics in the three preceding months. Swabs were sent at 4 °C to the lab and processed within 24 h.

This study was conducted in accordance with the ethical requirements, and the whole study protocol was approved by the Scientific committee of the Institute of Veterinary and Agricultural Sciences (Batna University, document n° 81/DV/ISVSA/UB1/2024).

### 4.2. Bacterial Isolation and Identification

Swabs were placed in Brain and Heart Infusion broth (BHI; Merck Millipore Merck KGaA, Darmstadt, Germany) and incubated aerobically at 37 °C for 24 h. One ml of each enrichment was plated on MSA Chapman agar (Merck KGaA, 64271 Darmstadt, Germany) and incubated at 37 °C for 24 h. One colony per morphology was picked up for further investigations.

Identification of the *Staphylococcus* genus was performed by PCR using primers detecting the genus-specific 16S rRNA sequence as described by Maes et al. [36]. Identification of the *S. aureus* and *S. pseudintermedius* species was performed by PCR using the *nuc* (for the species-specific identification of *S. aureus*) and *pse* (for the species-specific identification of *S. pseudintermedius*) primers, respectively described by Maes et al. and Sasaki et al. [37].

The *mecA* and *mecC* genes were systematically searched for by PCR using previously published primers [36,38].

### 4.3. Antimicrobial Susceptibility Testing

Antimicrobial susceptibility testing was performed using the disk diffusion method on Mueller–Hinton agar (BioRad, Marne-la-Coquette, France) according to the guidelines of the Antibiogram Committee of the French Society for Microbiology (CA-SFM, https://www.sfm-microbiologie.org/, last accessed on 1 September 2024). Results were interpreted using the clinical breakpoints referenced by the CA-SFM (https://www.sfm-microbiologie.org/2023/06/15/casfm-veterinaire-2023/ (accessed on 1 June 2024) for veterinary-related antibiotics and https://www.sfm-microbiologie.org/wp-content/uploads/2023/06/CASFM2023_V1.0.pdf (accessed on 1 June 2024) for human-related antibiotics). *S. aureus* ATCC 25923 was used as quality control. Fifteen antibiotics of veterinary and/or human interest were tested (Mast Diagnostics, Amiens, France) (Table S1).

### 4.4. Pulsed-Field Gel Electrophoresis

To assess the clonal relatedness, Pulsed-Field Gel Electrophoresis (PFGE) of *Sma*1-digested DNA was performed on all isolates belonging to the same staphylococcal species and originating from the same household. Genetic relatedness was assessed to identify duplicate isolates (when two identical isolates came from two different body sites of a single dog) or potential intra-household transmission. PFGE was performed under the following conditions: 14 °C at 6 V/cm for 24 h with pulse times ranging from 10 to 60 s and using an angle of 120°. DNA fingerprints were analyzed, and the dendrogram of patterns was made using the Dice correlation coefficient, with tolerance and optimization set at 0.5% and 1%, respectively (BioNumerics, Ghent, Belgium).

### 4.5. Short-Read Illumina Sequencing and Genomic Analyses

DNA was extracted using the NucleoSpin Microbial DNA extraction kit (Macherey-Nagel, Hoerdt, France). Library preparation and short-read whole-genome sequencing using Illumina NovaSeq6000 technology were outsourced (Eurofins Genomics, Ebersberg, Germany). Raw reads were quality trimmed to remove adapter sequences and poor-quality bases. The trimmed reads were used to de novo assemble genomes using Shovill v1.0.4. The quality of assemblies was assessed using QUAST v5.0.2 (Table S3).

Identification of resistance and virulence genes was performed on assembled genomes using the ResFinder v4.1 and VirulenceFinder v2.0.3 (with a minimum identity of 95%) tools from the Centre for Genomic Epidemiology (http://www.genomicepidemiology.org/ (1 June 2024)).

Genomic typing was also performed on assembled genomes using MLST v2.0.9 and *spa*Typer tools from the CGE.

### 4.6. Phylogenomic Analysis

A single-nucleotide-polymorphism (SNP)-based phylogeny was constructed separately for MRSA ST1, ST22, and ST80 isolates. The analysis was performed on genomes sequenced in the frame of this study, as well as on genomes of the same STs originating from Algeria, which were retrieved from the NCBI database [23,24,25]. For the phylogeny, the annotated assemblies (Prokka v1.14.6) were used as input for Roary v3.13.0. Recombination events were filtered using Gubbins v2.4.1, and SNP-distances were calculated using snp-dists v0.7.0. Phylogenetic trees were visualized with their metadata using iTOL v6, and SVG files were processed using InkScape v1.0 to guarantee the quality of the figures.

### 4.7. Statistics

To explore differences in the susceptibility patterns between *S. aureus* and *S. pseudintermedius* isolates, a chi-squared test was performed for all antibiotics tested. Proportions were considered as statistically different when *p* < 0.05.

## 5. Conclusions

Algeria is a country where possessing a pet dog has only been increasing for a decade. The question of resistant bacteria in these animals is thus under-reported, especially for *S. aureus* and *S. pseudintermedius*. This study identified a 14.8% proportion of both staphylococcal species, and 6.8% of the dogs were MRSA carriers. The identified clones belonged to the same STs as those circulating in hospitals in the country. Over the six isolates characterized, one carried the PVL and two the TSST toxin. Fortunately, no sharing of the *S. aureus* clone was observed between owners and their dogs. Nevertheless, our results highlight the need for a One Health approach to avoid the wider dissemination of such successful clones that are associated with resistance and virulence.

## Figures and Tables

**Table 1:** Resistance phenotypes of *S. aureus* and *S. pseudintermedius* from dogs.

	*S. aureus* (n = 26)		*S. pseudintermedius* (n = 17)		*p*-Value
	No.	%	No.	%	
Penicillin G	21	80.8	5	29.4	<0.05
Kanamycin	6	23.1	0	0.0	<0.05
Gentamicin	0	0.0	0	0.0	NA
Tobramycin	0	0.0	0	0.0	NA
Chloramphenicol	0	0.0	0	0.0	NA
Florfenicol	0	0.0	0	0.0	NA
Tetracycline	9	34.6	10	58.8	<0.05
Tigecycline	0	0.0	0	0.0	NA
Erythromycin	8	30.8	0	0.0	<0.05
Spiramycin	1	3.8	0	0.0	<0.05
Lincomycin	2	7.7	0	0.0	<0.05
Fusidic acid	2	7.7	1	5.9	0.131
Enrofloxacin	1	3.8	0	0.0	<0.05
Cefoxitin	8	30.8	ND	ND	NA
Cefovecin	ND	ND	0	0.0	NA
Linezolid	0	0.0	0	0.0	NA

NA: not applicable. ND: not determined. Cefoxitin is used as the marker of methicillin resistance in *S. aureus*, while cefovecin is used as the marker of methicillin resistance in *S. pseudintermedius*.

**Table 2:** Genomic characterization of MRSA isolates from dogs.

Strain	ST	*spa*-Type	Resistance Genes	SCC*mec*-Type	Virulence Genes	Replicon Types
64280	1	t127	*blaZ*, *mecA*, *ant(6)-Ia*, *aph(3′)-III*, *erm(C)*, *tet(K)*, *vga(A)V*	IVa(2B)	*hglABC*, *lukDE*, *seh*, *sak*, *scn*, *aur*, *splA*, *splB*	rep10, rep16, rep5a, rep7a, rep7c
64282	1	t948	*blaZ*, *mecA*, *ant(6)-Ia*, *aph(3′)-III*, *erm(C)*, *tet(K)*	IVa(2B)	*hglABC*, *lukDE*, *seh*, *sak*, *scn*, *aur*, *splA*, *splB*	rep10, rep16, rep5a, rep7a, rep7c
64292	1	t127	*blaZ*, *mecA*, *ant(6)-Ia*, *aph(3′)-III*, *erm(C)*, *tet(K)*	IVa(2B)	*hglABC*, *lukDE*, *seh*, *sak*, *scn*, *aur*, *splA*, *splB*	rep10, rep16, rep5a, rep7a, rep7c
64295	80	t639	*blaZ*, *mecA*, *ant(6)-Ia*, *aph(3′)-III*, *erm(C)*, *tet(K)*, *fusB*	IVc(2B)	*hglABC*, *lukDE*, *sak*, *scn*, *aur*, *splA*, *splB*, *edinB*, PVL	rep10, rep20, rep21, rep7c
64297	22	t845	*blaZ*, *mecA*	IVa(2B)	*hglABC*, *sak*, *scn*, *aur*, *seg*, *sei*, *sem*, *sen*, *seo*, *seu*, *tst*	rep20, rep5a
64314	7118	t223	*blaZ*, *mecA*	IVa(2B)	*hglABC*, *sak*, *scn*, *aur*, *seg*, *sei*, *sem*, *sen*, *seo*, *seu*, *tst*	rep20, rep5a

## Data Availability

The project was deposited in DDBJ/EMBL/GenBank under the BioProject accession number PRJNA1165664.

## Supplementary Materials

The following supporting information can be downloaded at: https://www.mdpi.com/article/10.3390/antibiotics13111090/s1, Table S1: Table of isolates; Table S2: SNP distance matrix; Table S3: WGS quality checks.
