# Supplementary material for: Methicillin-Resistant S. aureus Carrying the PVL and Toxic Shock Syndrome Toxin in Healthy Dogs in Algeria
Source: Antibiotics (Basel). 2024 Nov 15;13(11):1090. doi: 10.3390/antibiotics13111090 (PMC11590900; doi:10.3390/antibiotics13111090)
Supplement: Supplementary file 1 [file antibiotics-13-01090-s001.zip › TableS3_QC.pdf]

Table S3. WGS quality checks

Sample	FastQC	Quast				kraken/bracken	Sample
	Total Sequences_R2/R2	Total length	No. of contigs	N50	Coverage (X)	Identification	Abondance
64280	5935438	2816308	18	325943	636	*S. aureus*	0,90323
64282	5610595	2814920	21	326023	602	*S. aureus*	0,93939
64292	5743271	2814510	17	325979	616	*S. aureus*	1
64295	6395534	2831381	41	220293	682	*S. aureus*	0,98214
64297	5081790	2786135	38	132162	551	*S. aureus*	0,95312
64309	5919441	2718760	25	207780	658	*S. aureus*	0,97727
64314	5780321	2811846	40	127196	621	*S. aureus*	0,95312
